# The Establishment of the ϕ6 Genome Packaging Assay

**DOI:** 10.3390/v16010022

**Published:** 2023-12-22

**Authors:** Paul Gottlieb, Aleksandra Alimova

**Affiliations:** School of Medicine, The City University of New York (CUNY), New York, NY 10530, USA; aalimova@med.cuny.edu

This editorial describes the efforts to establish a genome packaging assay for the ϕ6 bacteriophage, which were performed in the laboratory of Leonard Mindich, Ph.D., at the Public Health Research Institute (PHRI), then located in New York City. It is best understood if read after the review in this Special Issue describing the discovery and classification of ϕ6. The author (PG) was a post-doctoral fellow in the laboratory, and was intimately involved in the research; hence, the story is told from the point of view of the primary participant. The successful development of the in vitro packaging methodology opened a path to further research, in particular the recombination and structural studies described in separate reviews also published in this Special Issue. The study described in this editorial follows the initial ϕ6 research described in the Special Issue section, “Discovery and Classification of the ϕ6 Bacteriophage: An Historical Review”. The reader is then advised to refer to “Heterologous RNA Recombination in the Cystoviruses ϕ6 and ϕ8: A Mechanism of Viral Variation and Genome Repair”, also in this Special Issue, for a contiguous discussion of the cytovirus studies. When the first phase of the classification of cystovirus ϕ6 concluded, the recombinant genetic technology had advanced, and complementary DNA (cDNA) synthesis and sequencing of the double-stranded RNA (dsRNA) could be accomplished [1,2]. However, the methodologies were not yet automated and, for the most part, had to be performed in each research laboratory. The absence of the mechanized facilities now commonly employed to quickly perform the sequencing procedures dictated that the studies required several years to complete. Therefore, the editorial review covers another decade, and begins with a description of the synthesis of the three cDNA segments, their expression of bacteriophage proteins, and the sequence analysis that identified the open reading frames (ORFs). The function of the bacteriophage proteins is described in “Discovery and Classification of the ϕ6 Bacteriophage: An Historical Review”; however, Table 1, describing the function and size of each protein, is added here for clarity. The protein copy numbers for the nucleocapsid proteins were determined by cryo-electron microscopy, while envelope proteins were only estimated by SDS gel analysis. The bulk of the genome classification work was performed in the laboratories of Leonard Mindich, Ph.D., at PHRI, and Helen Revel, Ph.D., of the Department of Molecular Genetics and Cell Biology at the University of Chicago. Both groups worked independently of each other, and there was considerable overlap of effort, but communication between each group was open, and could be described as “friendly competition” with an exchange of ideas, concepts, and materials.

In 1985, the laboratory of Helen Revel began their studies by determining the nucleotide sequences of the ends of the three ϕ6 genome segments L, M, and S [3]. The reasoning was that the requirements for genome packaging and replication were governed by these sequences, and the analysis also demonstrated that the 5′ ends of the m and s mRNAs were identical to the 5′ termini of the plus sense strands of the virion’s dsRNA. The enzymatic RNA sequencing methodology compelled base specific RNase cleavage reactions with the analysis in urea, based sequencing gels run at 800 to 1800 volts. All three dsRNA segments were found to have 18 homologous bases at the 5′ end, except for position 2, which is a U in the L segment and G in the M and S segments. As seen later, this difference was significant with regard to the temporal control of the bacteriophage transcription. The bacteriophage message RNAs are polycistronic, in contrast to the monocistronic mRNA of the reoviruses, and the next phase of the studies continued in both laboratories of Revel and Mindich, with the generation of cDNA clones and the in vitro gene expression. Two studies published approximately one year apart complement each other, with results that described the isolation of multiple fragments of the three dsRNA genome segments. The Mindich group was able to isolate cDNA fragments that recreated the entire M and S segments, and an approximately 1 kb piece of the L segment from the 5′ end. Using a coupled transcription translation assay, the in vitro synthesis of ϕ6 proteins helped to confirm the previous results based upon the S and M genetic maps. When the select cDNA segments were inserted into a shuttle vector that was able to replicate both in *Escheria coli* (*E. coli* HB101 and *Psedomonas phaseolicola*, *P. phaseoilicola* HB10Y), complementation of nonsense mutants previously located on the S and M segments was observed. The Revel research group was able to obtain cDNA fragments corresponding to the entire L segment, approximately 98% of the S segment, and approximately 67% of the M segment, constituting a library of about 20 cDNA pieces. Proteins derived from ϕ6 were synthesized in minicells, and the order of the genes on the L segment was established as 7, 2, 4, and 1. The synthesis of cDNA enabled the establishment of a limited restriction endonuclease map calculated from the cDNA sequence.

**Table 1:** Major structural proteins of cystovirus ϕ6, its molar weight, number of copies per virion, and basic cell functions. Integral membrane protein copies have not been determined.

Protein	Molecular Weight, KDa [4]	Copies per Virion [5]	Function
P1	93.0	120 or 60 dimers	Major structural protein
P2	74.8	12	RNA-directed RNA polymerase
P4	35.0	72 (12 hexamers)	NTPase packaging motor, transcription
P7	17.2	60	Assembly cofactor; packaging cofactor
P5/P11	24.0		Lytic endopeptidase; murein peptidase
P8	15.8	600 (200 trimers); 720	Shell
P6	21.0		Integral membrane protein
P3	84.0		Binding protein
P9	9.5		Major envelope protein;
P10	6.0		Putative holin protein
P12	20.3		Envelope acquisition (nonstructural)
P13	7.6		Integral membrane protein
P14	6.8		Integral membrane protein

The next aim of the research was the sequence analysis of the entire bacteriophage genome; this research was primarily performed by the Mindich laboratory (Figure 1, S: NC_003714, M: M17462, L: M17461). Due to the technological limitations at the time, the entire sequence analysis of the three-dsRNA genome took two years to complete. The sequence analysis preceded and ultimately guided the bacteriophage assembly studies, and therefore warrants discussion in some detail. The sequencing of each segment utilized synthesized cDNA fragments first described in Mindich et al., 1985 [6] which, together, encompassed the entire S and M segments (later additional cDNA copies of the L segment had to be isolated to obtain a library of the entire L). For the most part, the S nucleotide sequence analysis utilized the method of Maxam and Gilbert, while the M and L analysis was performed only with dideoxy chain termination (now known as the Sanger dideoxynucleotide chain-termination method). The sequence studies, when completed, confirmed the earlier conventional mutational analysis observations with regard to gene order, and the polar relationships between genes 8, 12 and 9, 5 (11). For example, it was clearly seen that translational coupling between gene 8 and gene 12 was the consequence of the P8 encoding mRNA stop UAA overlapping with the P12 mRNA start AUG and, additionally, gene 12 lacked a properly spaced Shine–Dlgarno (SD) sequence that compelled ribosome loading on the P8 mRNA. The same polar arrangement was found on the L segment between genes 7 and 2, in that the gene 7 mRNA stop codon overlapped the P2 mRNA start codon and gene 2 lacked an evident SD sequence. These two observations also explained why, quantitatively, there is less protein P12 synthesized than P8, and much less RNA polymerase P2 than P7, as the ribosome readthrough efficiency is low on the second linked protein-encoding mRNA. Translational coupling was also observed between the intrinsic membrane protein P9 and murein peptidase P5/11, in that the distal gene has no evident SD site to initiate P5/11 translation. Additionally, of great interest is nature of P5 and P11, requiring the “backslash” identification of the two proteins as P5/11. The P11 protein was observed and identified only in rifampin treated cell lysates infected with ϕ6, but never isolated and purified from a bacteriophage, therefore is considered a non-structural protein. On the M dsRNA segment, the curious reciprocal polarity between the genes that assemble the host attachment apparatus, P6 and P3, was not explained, and this still remains a question that requires an answer. Earlier explanations (proposed when the polarity was first noted by the classical genetic studies) postulated cumbersome infoldings of the gene 6 and 3 carrying mRNA region, and this looping of the mRNA alternatively blocked the P6 or P3 start codons. This extremely unlikely hypothesis was quickly discarded (comment of LM to PG, approximately 1988.) P6 has a significant hydrophobic profile supporting the notion that it is integral to the bacteriophage membrane anchoring P3 on the bacteriophage envelope. In 1994, the laboratory of Helen Revel found gene 14 upstream of gene 7, and the encoded P14 protein has a molecular weight of 6.8 kDa (Figure 1). When an amber nonsense mutation was inserted in the gene and phage stocks were isolated, these exhibited slightly smaller burst sizes in several host strains. Yet, while no discrete function could be assigned to the protein, it was speculated that P14 stimulates translation of early genes.

Additional identity among the three segments was evident from the sequence studies extending the observations of Iba et al. (1982). Identical regions located at the 5′ ends with 12 bases have implications for temporal transcription regulation (Figure 1 and Figure 2). The 3′ ends of each segment have a longer similarity region extending 80 nucleotides, but include several gaps. The plus sense 3′ ends would later be shown to have extensive identical secondary structures serving as initiation sites for minus strand replication. When the amino acid sequence of protein P4 was examined, it showed similarities to a variety of proteins, with functions ranging from UV repair to bacterial transport, but not RNA polymerases. Since the protein was already understood to be a component of the procapsid (PC), the study discussion proposed that it played a role in RNA packaging. Four years later, when the protein was recognized to contain a nucleotide-binding site (referred to as the Walker Motif) and exhibited nucleoside triphosphate phosphohydrolase activity, the idea was considerably strengthened.

Once the cDNA pieces derived from each dsRNA segment were synthesized and a complete restriction endonuclease map was created the cDNA representing entire genomes was inserted in expression plasmids after the *lac* promoter. The initial task was the production and isolation of a polyhedral particle or PC from *E. coli*. Plasmids containing the L segment of ϕ6 were transformed into *E. coli*, and the cell cultures were induced with IPTG at 26 °C. The gradient-isolated particles from the cell lysate were approximately 130S, which is reasonably close to the 120S particle from ϕ6 infected *P. phaseolicola*. SDS-PAGE gel analysis confirmed that the P1, P2, P4, and P7 proteins were expressed, and their stoichiometry was in the same proportion as those found in the bacteriophage PC. Multiple constructs were created to study the relationship between protein composition of the PC, including deletions to create P1 only, P1+P4, P1+P2, and P1+P7 particles. The complete particles, as well as particles consisting of only P1 and P4 (produced by cDNA deleted of genes 2 and 7), were stable in gradients, in contrast to extremely unstable P1, P1+P2, and P1+P7 particles. In the absence of P1, no particles formed. A P7+P2 lysate applied to a P1+P4 lysate reconstituted a complete particle. Negative stained samples studied by transmission electron microscopy showed that the complete particles had a polyhedral shape, and many also appeared in star or 10-knob necklace conformations that, in the projection images, were suggestive of a dodecahedron (Figure 3A). Electron micrographs of thin sections of *E. coli*-producing PCs showed very large numbers of polyhedral particles inside the cells, which appeared to be in the process of lysing (Figure 3B). Building upon the initial reconstitution effort in the Mindich laboratory, extensive in vitro assembly kinetic studies were later performed in Helsinki.

The next goal was the development of an in vitro RNA packaging and replication assay that utilized the empty recombinant PCs isolated from *E. coli.* (Those so inclined to read the original paper might note that the first word in the Introduction is “bacteriophate”, not the proper spelling “bacteriophage”. This was in 1990, and copy proofs were still corrected by hand with pen or, pencil and author PG assigned to the task edited with diligent accuracy—that is, except for the very first word of the text). As described in “Discovery and Classification of the ϕ6 Bacteriophage: A Historical Review”, the overall features of the ϕ6 assembly and RNA synthesis mechanism were recognized, but greater resolution of the mechanisms was required, and the in vitro system facilitated it. In particular, the characteristics unique to packaging of segmented dsRNA viruses could now be elucidated. For example, the bacteriophage particles contain only one each of the three genomic segments, and the efficiency of plating is approximately one, implying an accurate RNA recognition and counting mechanism. The packaging signals of each RNA segment were identified, and the order of packaging determined as s, m, and l (Figure 4).

The initial step was the isolation of ϕ6 mRNA precursors to demonstrate RNA polymerization activity and packaging, and the quickest approach was to use transcripts from the actual NCs by the method described by Emori et al. Single-stranded mRNA was rapidly isolated on a cellulose column, using elution buffer with 20% ethanol. The typical reaction mixture included ^32^P-labeled UTP, no more than 2 μg of empty PC, approximately 1 μg of RNA, and ran at 27 °C for 90 min. Both magnesium and manganese ions were essential for the reaction. This simple assay method demonstrated several fundamental qualities of the ϕ6 PC. The reaction was capable of synthesizing labeled dsRNA, and this product showed the same gel migration rate as the normal ϕ6 dsRNA. Labeled plus and minus strands were identified using strand separating gels, as described by Pagratis, and Revel consistent, with the mechanism that first a minus strand was synthesized from the positive mRNA template, and then stand displacement produced additional positive sense RNA. Therefore, the newly formed dsRNA was capable of supporting the transcriptase function of the polymerase in which the minus and plus strands RNA products were analyzed on a strand separating gel. The PCs lacking P2 did not have activity, and those without P7 had extremely low activity.

Years later, during cryo-electron microscopy studies of the PC, a mild controversy arose between the Mindich laboratory and the then-independent Gottlieb laboratory regarding the positioning and function of the P2 and P7 proteins in cystoviruses. Within empty PCs, P2 and P7 occupy the inner three-fold axis, either as mutually occluding binding sites, as shown by the Mindich group, or can both reside at the same position, which was the Gottlieb group’s opinion. After packaging and RNA replication, the Gottlieb group expected that the P2 is relocated to the inner 5-fold axis of the NC directly beneath the hexameric NTPase packaging portals, in “anticipation” of initiating transcription upon host cell infection. This subject remains open to additional study, and is described in detail in “The Cystovirus Bacteriophages: Dynamic Interactions during Capsid Maturation” (which is linked to this Special Issue).

In the following study, using the in vitro packaging assay, the Mindich laboratory confirmed that the preformed ϕ6 PC has the ability to select and package the three bacteriophage mRNA segments vs. the alternative model, where a capsid forms surrounding the genomic nucleic acid. The packaging reaction was found to be dependent on the presence of ATP (or even dATP), anticipating the future discovery of the NTPase activity of the P4 hexamer, which propelled mRNA into the PC. The study demonstrated that packaging of the ssRNA was independent of final dsRNA replication, and once internalized within the filled PC, ssRNA is resistant to RNAase digestion. Two additional studies were completed in the first half of 1992, which further defined the mechanisms for mRNA selection with regard to the dependence of RNA replication on full packaging of the three ssRNA segments. Replication reactions were performed using combinations of ssRNA segments and, this time, the precursor RNA was produced from in vitro transcripts directed by recombinant plasmids containing the T7 promoter, greatly simplifying the preparation of the required nucleic acid reagents. The two studies confirmed that minus strand synthesis was dependent on the complete packaging of the three mRNA segments. The complete packaging and minus strand synthesis initiation could be triggered, even if only a short 5′ end segment completed the genomic set. The truncated segment (whether deleted s, m, or l) could not support replication of its own minus strand itself, but the P2 polymerase became capable of replication of the other two. The notion of Gottlieb et al. that any of the three mRNA segments could be integrated into the PC in any order suggested that three specific binding sites had to exist on the PC, and each genomic precursor has a unique packaging signal. However, later efforts by the Mindich laboratory, along with structural studies by the Bamford laboratory, in Helsinki, and the Steven laboratory, at the National Institutes of Health (Bethesda, MD, USA), clearly showed that there is a sequential packaging order of s, m, and l RNA, accompanied by three stages of expansion by the PC that reveal each segment’s binding site.

The next step in the research was the identification of the packaging-specific sequences of each of the mRNA segments. T7-directed transcription plasmids carrying cDNA copies of the three genome segments were open near the 5′ end and digested with endonuclease *Bal 31* for varied reaction times to select internal deletions of increasing size. Subsequently, the T4 polymerase treatment insured blunt ends post *Bal 31* digestion, and ligation of the ends completed the constructions. Effective packaging of a particular strand was scored by its serving as a template for its own minus strand synthesis and stimulus of synthesis of the other two packaged segments. The method provided the first approximation of the packaging sequences that were estimated to be 250, 300, and 205 nucleotides for s, m, and l, respectively, and these genome regions are indicated in Figure 1. An initial effort at folding the regions using the mfold program of Zuker suggested the presence of a secondary structure, but no similarity among the three RNA segments was noted. Details of the secondary structure were determined in future research, and were ultimately found to be highly structured, as shown in Figure 5 (the s segment pac region), reproduced from Hanhijarvi et al., 2016 [9]. Ultimately, adaptation of the ϕ6 packaging assay, along with the use of an optical tweezers method, demonstrated the packaging rate, showing that this proceeds in intermittent slow and fast phases governed by the ssRNA unfolding rate of the complex secondary structure.

The establishment of an in vitro packaging assay, and initial analysis of the bacteriophage mRNA signaling sequences for both packaging and replication, opened the path for further research on rescue, recombination, and structural studies, as described in the Special Issue articles, “Heterologous RNA Recombination in the Cystoviruses ϕ6 and ϕ8: A Mechanism of Viral Variation and Genome Repair, Structural Studies on the Bacteriophage ϕ6 Capsid and its Transformation During the Virus Life Cycle” and the linked review “RNA Packaging in the Cystovirus Bacteriophages: Dynamic Interactions during Capsid Maturation”. Therefore, this review illuminates the research and path that led to recent and ongoing studies of cystovirus assembly covered in the next reviews in this Special Issue.

## Figures and Tables

**Figure 1:**
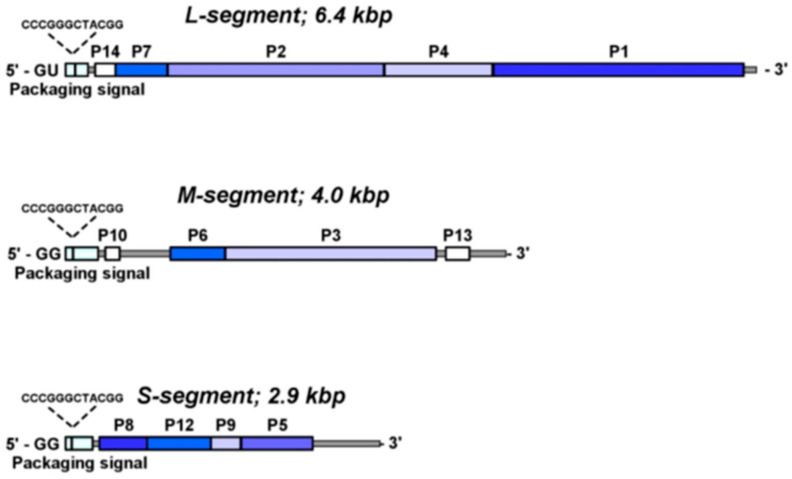
Genetic map of the three ϕ6 genome segments. The coding regions for structural proteins are highlighted in different shades of blue, and the coding regions for non-structural proteins are in white. The packaging signal regions are indicated as cDNA sequence format.

**Figure 2:**
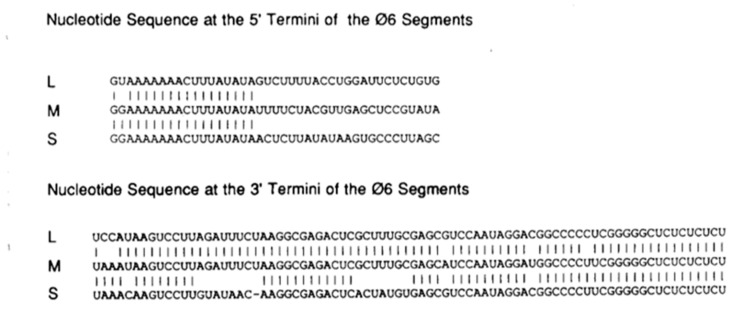
Nucleotide sequences at the termini of the three genomic segments of ϕ6. The plus strands are shown and vertical lines indicate identity. Reproduced from Mindich et al., 1988 [7].

**Figure 3:**
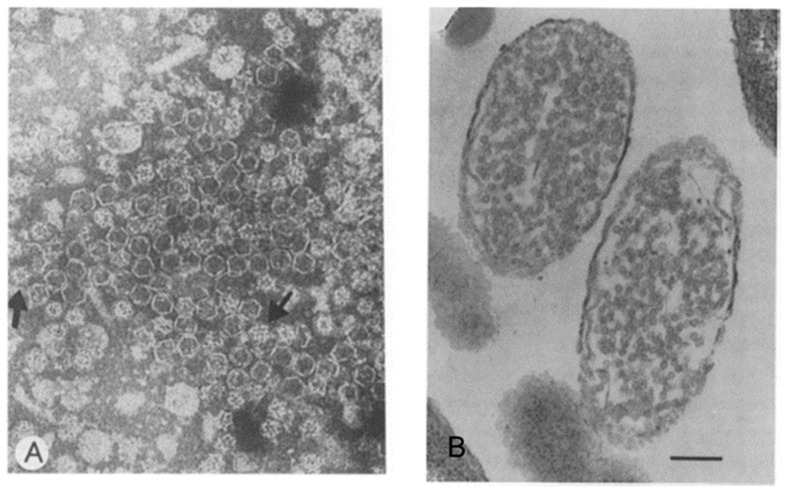
Transmission electron micrographs of negatively stained samples (**A**). Recombinant PCs isolated from *E. coli*. The estimated diameter of the PC is approximately 50 nm, and arrows indicate the “star” conformation. (**B**). Electron micrograph of a section of *E. coli* filled with assembled recombinant PC. Bar, 200 nm. Reproduced from Gottlieb et al., 1988 [5].

**Figure 4:**
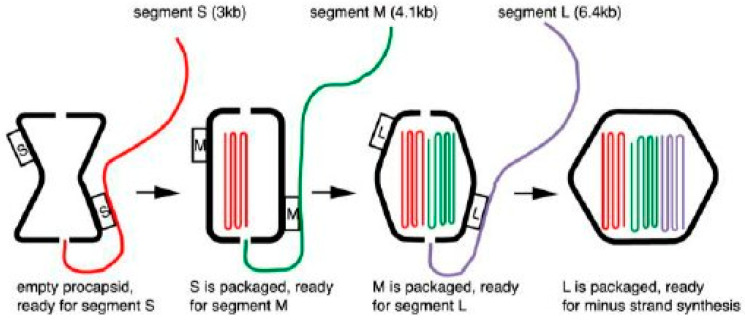
Sequential packaging model of the three single stranded RNA segments in the order s, m, and l. After each segment enters the PC, a new RNA pac site recognition signal is revealed. Reproduced from Mindich, L. (2004) [8].

**Figure 5:**
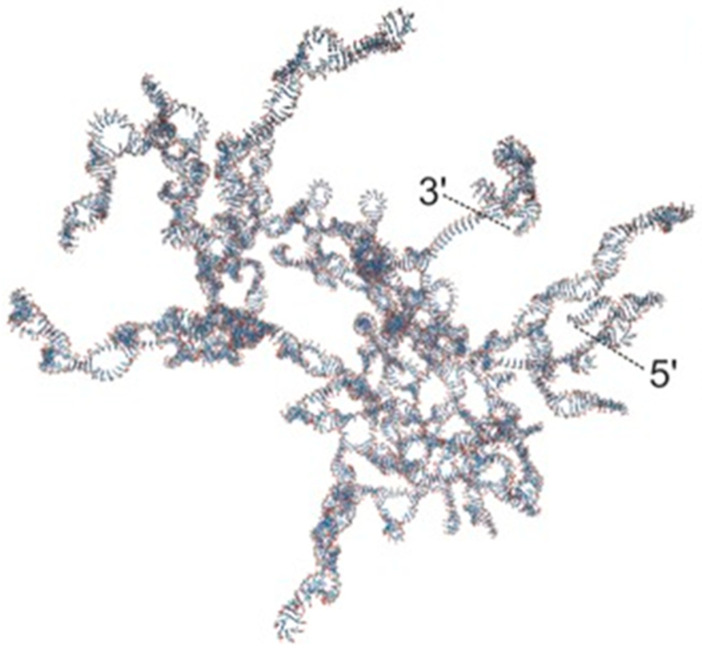
S segment, 5′ end mfold, reproduced from Hanhijarvi et al., 2016 [9].
